# A Retrospective Analysis of Intervention for Testicular Torsion: Searching for a Hallmark of High Reliability

**DOI:** 10.1097/pq9.0000000000000232

**Published:** 2019-12-16

**Authors:** Jennifer K. Calder, Bennett W. Calder, Chase A. Arbra, Robert A. Cina

**Affiliations:** *Department of Pediatrics, Division of Pediatric Emergency Medicine, Medical University of South Carolina, Charleston, S.C.; † Department of Surgery, Division of Pediatric Surgery, Medical University of South Carolina, Charleston, S.C.

## Abstract

Consistent delivery of high-quality care is a marker of health-care system reliability. Although clinically abstracted outcome databases have revolutionized surgical quality improvement efforts for many high-volume procedures, their utility in aiding the improvement of time-sensitive processes is less clear. The purpose of this study was to determine whether process measures surrounding the delivery of timely surgical care could delineate the variability in the outcome of patients with testicular torsion. Our secondary aim was to use the data to drive quality improvement efforts locally.

**Methods:**

We performed a retrospective review of encounters for testicular torsion in patients less than 18 years. Characteristics of patients undergoing detorsion/orchiopexy and orchiectomy were compared. We evaluated orchiectomy as a function of age, insurance status, time of presentation, duration of symptoms, time to ultrasound, and time to surgery.

**Results:**

Over 10 years, we identified 46 patients, of whom 21 met inclusion criteria. Twelve patients (57.1%) underwent detorsion/orchiopexy, whereas 9 patients underwent orchiectomy. After-hours presentation and age were not predictive of orchiectomy, whereas the duration of symptoms was predictive of orchiectomy. Differences in time to surgery for orchiopexy and orchiectomy groups approached but did not reach significance (*P* = 0.07).

**Conclusions:**

Significant variability persists in the timely delivery of comprehensive and coordinated care for children suffering from time-sensitive surgical conditions. Pediatric surgical programs would realize significant benefit from a surgical quality improvement program that incorporates validated process metrics, along with outcome measures, to help drive efficiencies and integrate care more effectively.

## Abstract 2

Supplemental Digital Content is available in the text.

## INTRODUCTION

At the turn of the century, the Institute of Medicine set forth goals for the delivery of care in the 21st century: safe, effective, patient-centered care that is equitable, efficient, and timely.^1^ Since that time, we have witnessed an increasingly robust set of tools by which to improve the care of the patents who place their lives in our hands. The National Surgical Quality Improvement Program, which was developed in the 1990s in the Veterans Health Administration Hospitals, and later expanded to the private sector under the guidance of the American College of Surgeons, has arguably led to one of the greatest improvements in the care of surgical patients in the United States. Hospitals and health systems participating in this clinically abstracted database have demonstrated both improved surgical outcomes^2^ and decreased health-care expenditures for complications.^3^

A pediatric version of the National Surgical Quality Improvement Program was piloted in 4 hospitals in 2008^4,5^ and has since expanded to 109 sites across the United States. Using this data source, dovetailed with a structured quality improvement (QI) methodology, we have witnessed the power of an accessible, robust quality outcomes database on not only our local outcomes but also the lives of our patients. Although the quality of surgical care for high-volume procedures certainly benefits from direct outcome measurement, we were concerned that this system did little to inform the “near misses” often seen in the time critical care of emergent and urgent surgical conditions.

Patients requiring emergency surgery are particularly prone to delay whether appropriate systems are not in place to accommodate the additional resource burden these procedures require.^6^ For these types of conditions, an evaluation of the process is equally necessary to the evaluation of outcome in ensuring expeditious workup and timely treatment. Although there are a variety of surgical conditions that could potentially benefit from this approach, they all share a common denominator: time to treatment determines tissue salvage.^7^ This adage is particularly true for patients with testicular torsion, where necrosis and the need for orchiectomy increase with the duration of testicular symptomatology.^8^ Although decreasing time to diagnosis and treatment are important factors in the care of these patients, an evidence-based timeline goal, such as those described for the “sepsis shock clock,”^9^ may help to define operational goals and set a benchmark toward which to aim.

In this study, we sought to find a hallmark for reliability and consistency in the delivery of these time-sensitive procedures. Specifically, using testicular torsion as a model, we aimed to (1) examine variability in the diagnosis and surgical management of this time-sensitive condition; (2) identify an institutional time standard for management of pediatric testicular torsion; (3) identify deficiencies in the care of these patients specifically to guide ongoing QI efforts. We hypothesized that in patients presenting with this relatively infrequent diagnosis, time to diagnosis, time to treatment, and after-hours presentation would correlate positively with orchiectomy rates and inversely with testicular salvage (TS) rates.

## METHODS

A retrospective chart review was performed at the Medical University of South Carolina to identify patients less than 18 years of age with acute testicular torsion. We obtained Institutional Review Board approval before acquiring a list of patients or collecting any data. The Medical University of South Carolina clinical data warehouse was queried for patient charts from 2005 to 2015 with torsion-associated ICD-9/10 (International Classification of Diseases) diagnosis and/or CPT (Current Procedural Terminology) procedure codes, including 608.2, N44.0, N44.01, N44.02, N44.03, N44.04, 54520, 54600, 54640, 54650, 54692, 44055, 43194, and 43215. Manual chart review was performed to verify the diagnosis of acute testicular torsion with a primary presentation to our institution. We excluded patients with intermittent torsion, neonatal torsion, and chronic testicular torsion. Those patients who presented to an outside institution and received diagnostic imaging before transfer were also excluded.

Data were collected on patient demographics, date/time of arrival, date/time of imaging, time of day of presentation (after-hours versus normal daytime hours), type of imaging utilized, total duration of symptoms before presentation, clinical presentation, date/time of surgery, operative findings, and type of surgery performed (orchiectomy or detorsion/orchiopexy). We defined normal daytime hours from 7 am to 5 pm Monday–Friday. We then divided patients into groups based on the surgery they received, orchiectomy versus detorsion/orchiopexy. Statistical analysis was performed to identify differences in time delay to diagnosis and time delay to therapeutic intervention between groups. Statistics were performed using SPSS software (IBM Analytics, Armonk, NY). We used descriptive and univariate analyses for demographic variables. The distribution of proportions was compared using Chi-square analysis. Group diagnostic times, treatment times, and the total duration of symptoms were compared using unpaired Student’s *t* tests. We placed factors that approached statistical significant (*P* < 0.1) on univariate analysis in a multivariate binary logistic, forward conditional, regression model.

## RESULTS

**Fig. 1.**
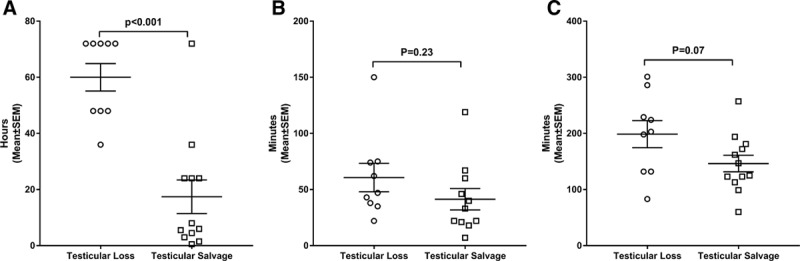
Comparison of duration of symptoms (A), time for presentation to diagnostic Imaging (B), and time from presentation to operative exploration (C) between patients experiencing TS and TL.

We identified 46 pediatric patients over 10 years with the above-listed ICD and CPT codes. We excluded 25 patients for intermittent torsion (60%), neonatal or perinatal torsion (12%), chronic torsion with complete infarction (8%), presentation and imaging at outside institution (4%), incomplete data points (4%), or other (including ambiguous genitalia, 12%). We included 21 patients in the analysis with a median age of 13.6 years. Twelve patients (57.1%) underwent detorsion and orchiopexy (TS), whereas 9 patients (42.9%) required orchiectomy [testicular loss (TL)]. There were no significant correlations between age, race, insurance status, and after-hours presentation between those patients who experience TS versus TL (Table 1). The mean duration of symptoms (Fig. 1A) before the clinical presentation was 17.4 ± 20.7 hours in those patients experiencing TS, which was significantly shorter than 60.0 ± 14.7 hours in the TL group (*P* < 0.001).

**Table 1.**
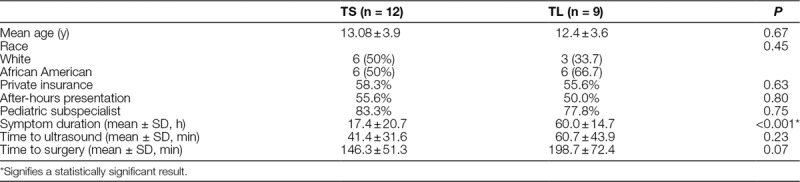
Patient Demographics and Case Details

Twenty patients (95.2%) underwent an ultrasound before surgery. One patient who did not undergo imaging proceeded directly to the operating room for an orchiopexy and TS. The average time delay from presentation to ultrasound was longer for the orchiectomy patients (60.7 minutes) than the orchiopexy group (41.4 minutes) by 19.3 minutes (*P* = 0.23) but did not achieve statistical significance (**See figure 1B, Supplemental Digital Content 1**, which shows the time for presentation to diagnostic imaging http://links.lww.com/PQ9/A142). The time delay in minutes from presentation to operative exploration (TS 146.3 ± 51.3 minutes versus TL 198.7 ± 72.4 minutes) approached but did not reach statistical significance (*P* = 0.07) (**See figure 1C, Supplemental Digital Content 2**, which shows the time from presentation to operative exploration for patients experiencing TS or TL, http://links.lww.com/PQ9/A142).

**Fig. 2.**
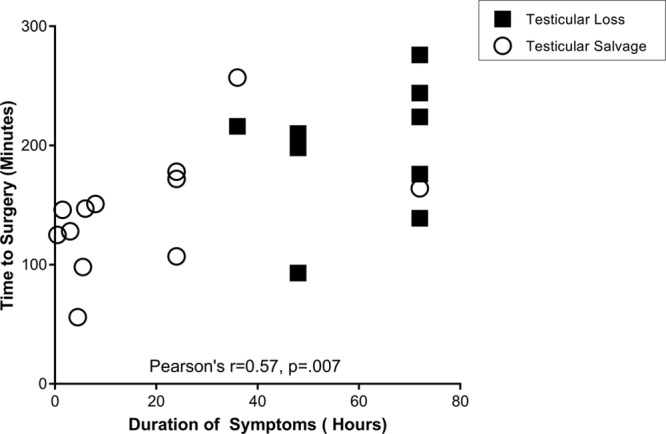
Relationship of the duration of symptoms vs time to surgery.

**Fig. 3.**
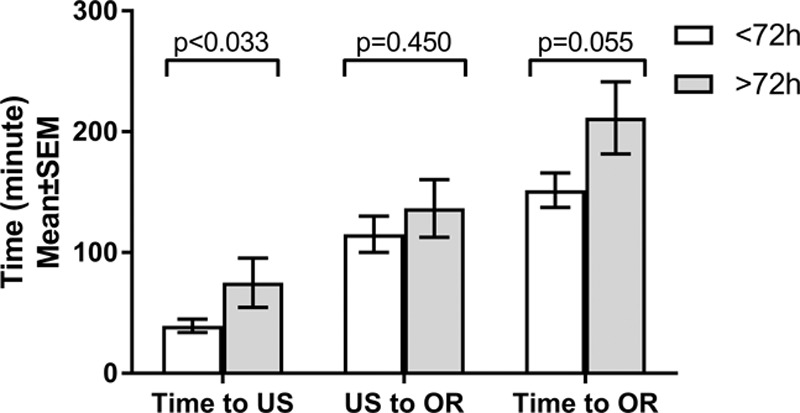
Time measures based on the duration of presenting symptoms.

Multivariate logistic regression analysis of patient and encounter-specific variables did not identify additional factors affecting orchiectomy or TS outcomes. After adjusting for the time delay to surgery, the total duration of symptoms was the only variable associated with TL to reach statistical significance (*P* = 0.016). There was a significant correlation (Pearson’s *r* = 0.568, *P* = 0.007) between the total duration of symptoms and the delay to surgical exploration (Fig. 2). When grouped by the duration of symptoms, patients presenting with shorter duration of symptoms experienced significantly shorter times to imaging, and a trend toward shorter times to definitive operative management (Fig. 3). The after-hours presentation was not associated with decreased access to timely imaging or operative intervention (**See table, Supplemental Digital Content 3**, which summarizes the effect of time of presentation on timely management, http://links.lww.com/PQ9/A143).

## DISCUSSION

The recent development of clinically abstracted outcome databases has empowered surgical programs to address many facets of patient care through standardization, persistent mindfulness for safety, and multidisciplinary organizational teamwork. Despite these advances, outcome data are less helpful in guiding efforts seeking to refine the efficiency of care provided by integrated systems. The addition of process metrics has been proposed to aid in streamlining care between the multiple teams (emergency department, radiology, and surgery) required to achieve an optimal outcome. By evaluating process measures surrounding male gonadal torsion, we identified significant variability and were able to highlight areas of opportunity within our system to inform process improvement efforts.

Consistent with other reports, the greatest contributing factor to gonad loss was the duration of symptoms at presentation.^8,10^ Although this may be modifiable with increasing primary care provider and patient education, there were significant hospital factors identified following presentation as well. Timely access to ultrasound (<30 minutes from presentation) was possible for roughly a third of patients. Unfortunately, nearly an equal number waited for imaging greater than 60 minutes, with 1 patient experiencing a 150-minute delay. These results highlight the need to establish the role of point-of-care ultrasound in the diagnostic workup of the acute scrotum.

The time required to transport the patient to the operating room (OR) and mobilize the staff was also highly variable, with a mean duration of 120 minutes. Gonadal torsion is a level 1b case in our OR triage system to have the patient in an operating room in 30–60 minutes from posting (time of operative decision), a metric achieved in only 15% of our patients. Delay from the time of presentation to therapeutic intervention did not reach statistical significance with a *P* value of 0.07. It is important to note that this value, calculated during the analysis of a relatively small patient population, suggests that increasing the power of the study might achieve significance.

We identified a rather unusual relationship between the total duration of symptoms at presentation with time to surgical exploration. This observation appears to be driven not only by a statistically increased time to ultrasound but also longer wait times for surgical therapy. These phenomena may be due to either (1) a difference in patient presentation (intermittent torsion with an indolent presentation) or (2) bias of the treating physicians who believe gonadal salvage is not possible due to the prolonged symptomatology, and therefore do not expedite time to intervention. The investigation into the underlying cause of this with our team reveals that both factors are likely involved.

### Process Optimization

Optimal outcomes for testicular torsion are predicated on rapid identification of the condition at triage, timely diagnosis by ultrasound, and urgent surgical exploration. We had hypothesized that urgent evaluation and surgical therapy would be statistically longer in a patient presenting at night and on weekends. The data disproved this theory in which we found nonstatistically shorter times to diagnosis and surgical exploration in these patients. The delay experienced by patients presenting during normal business may be due to the constraints of a system that operates at or near capacity with scheduled imaging and operative cases. We are currently exploring the feasibility of creating a dedicated pediatric operating room for emergency surgery. This approach has been found to have a significant effect on operating room access^6^ as reported by other groups.

**Fig. 4.**
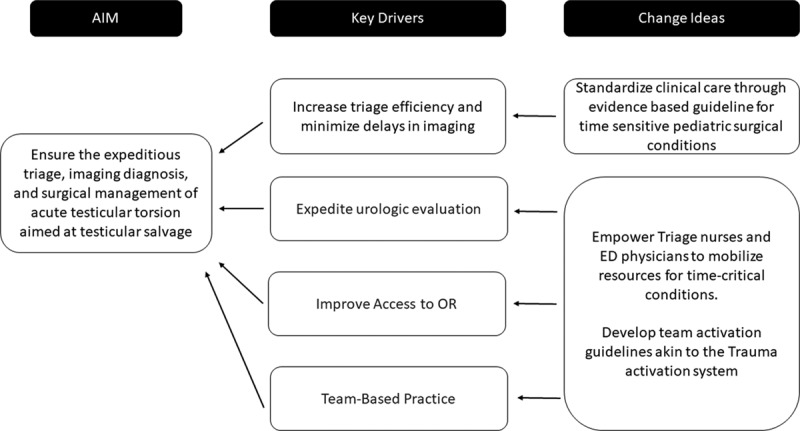
Key driver diagram for process improvement.

A specific aim and key driver diagram to inform a QI team are illustrated in Figure 4 to help guide our future efforts. The QI team should aim to decrease the time from symptom onset to surgical intervention from a baseline of 35 to 20 hours over a 1-year period by implementing interventions to impact the key drivers of (1) efficient triage and imaging, (2) expeditious urology consultation, (3) optimal access to the operating room, and (4) team-based practice. These drivers promote rapid communication among providers, coordination of care, and efficient use of necessary resources. To successfully optimize these key drivers, an evidence-based program, such as TeamSTEPPS 2.0, should be utilized to cultivate the multidisciplinary team. Developed by the Agency for Healthcare Research and Quality and the Department of Defense, the structured TeamSTEPPS system will allow our providers to collaborate and communicate more effectively through an evidence-based methodology. This program has a proven track record in enhancing the delivery of care in time-sensitive environments such as the emergency department.^11^

The requirements for the optimal care of patients with gonadal torsion, button battery ingestion, or any other time-sensitive procedure are not different from those required for the management of an acutely injured trauma patient. The trauma activation model that is predicated on a standardized approach to work up, communication, resource mobilization, and timely communication with operating room staff may be the ideal platform to emulate. We are currently exploring the feasibility of such a system within our hospital.

### Limitations

This study suffers from the shortfalls inherent to a retrospective design and the small numbers of patients experiencing this relatively infrequent condition. A prospective, multi-institutional study would yield significantly more actionable data and allow for risk model creation. Data derived from these models could inform a robust process improvement methodology, thereby increasing the efficiency of care for patients and potentially avoiding morbidity due to delays in care. Nonetheless, we feel that this study has significantly aided us in defining the problem of variability within our system and highlights areas of opportunity for improvement in team building, communication, and system integration.

## CONCLUSIONS

Significant variability persists in the timely delivery of comprehensive and coordinated care for children suffering from time-sensitive pediatric surgical conditions. Pediatric surgical programs would realize significant benefit from a surgical QI program that incorporates validated process metrics, along with outcome measures, to help drive efficiencies and integrate care more effectively.

## ACKNOWLEDGMENTS

Mentor: Robert A. Cina, MD, Associate Professor of Surgery. This research received no specific grant from any funding agency in the public, commercial, or not-for-profit sectors.

## DISCLOSURE

The authors have no financial interest to declare in relation to the content of this article.

## Supplementary Material
