# Supplementary material for: A Retrospective Analysis of Intervention for Testicular Torsion: Searching for a Hallmark of High Reliability
Source: Pediatr Qual Saf. 2019 Dec 16;4(6):e232. doi: 10.1097/pq9.0000000000000232 (PMC6946220; doi:10.1097/pq9.0000000000000232)
Supplement: Supplementary file 1 [file pqs-4-e232-s001.docx]

Table 2. Effect of Time of Presentation on Timely Management.

|  | Daytime | After Hours | p-value |
| --- | --- | --- | --- |
| Time to US (mean±sd) | 56.6±48.2 | 44.7±20.2 | 0.46 |
| Time to OR (mean±sd) | 184.9±60.1 | 154.1±68.9 | 0.29 |
