# Supplementary figures and images for: A Retrospective Analysis of Intervention for Testicular Torsion: Searching for a Hallmark of High Reliability
Source: Pediatr Qual Saf. 2019 Dec 16;4(6):e232. doi: 10.1097/pq9.0000000000000232 (PMC6946220; doi:10.1097/pq9.0000000000000232)

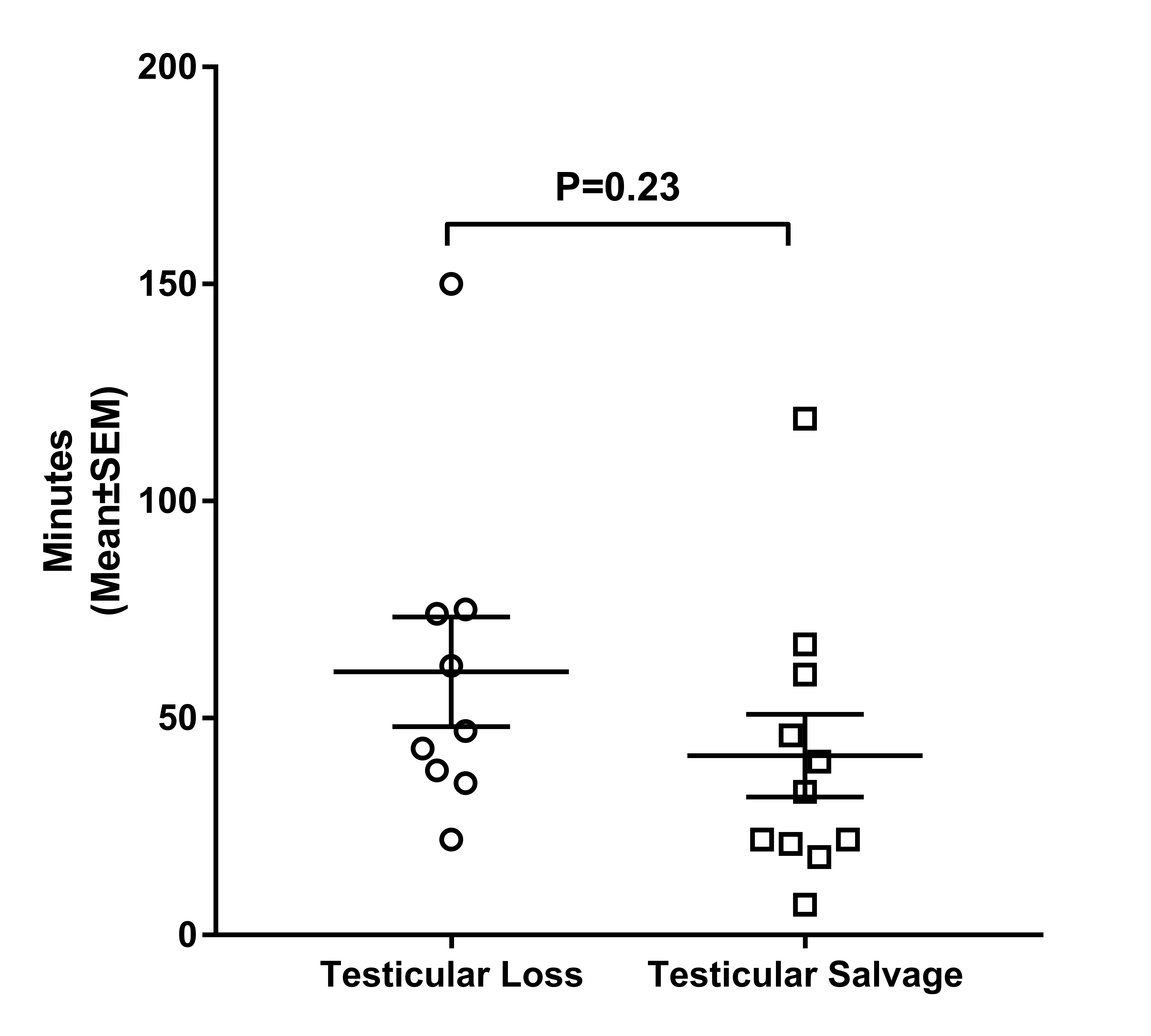

Supplement: Supplementary file 2 [file pqs-4-e232-s002.tif]

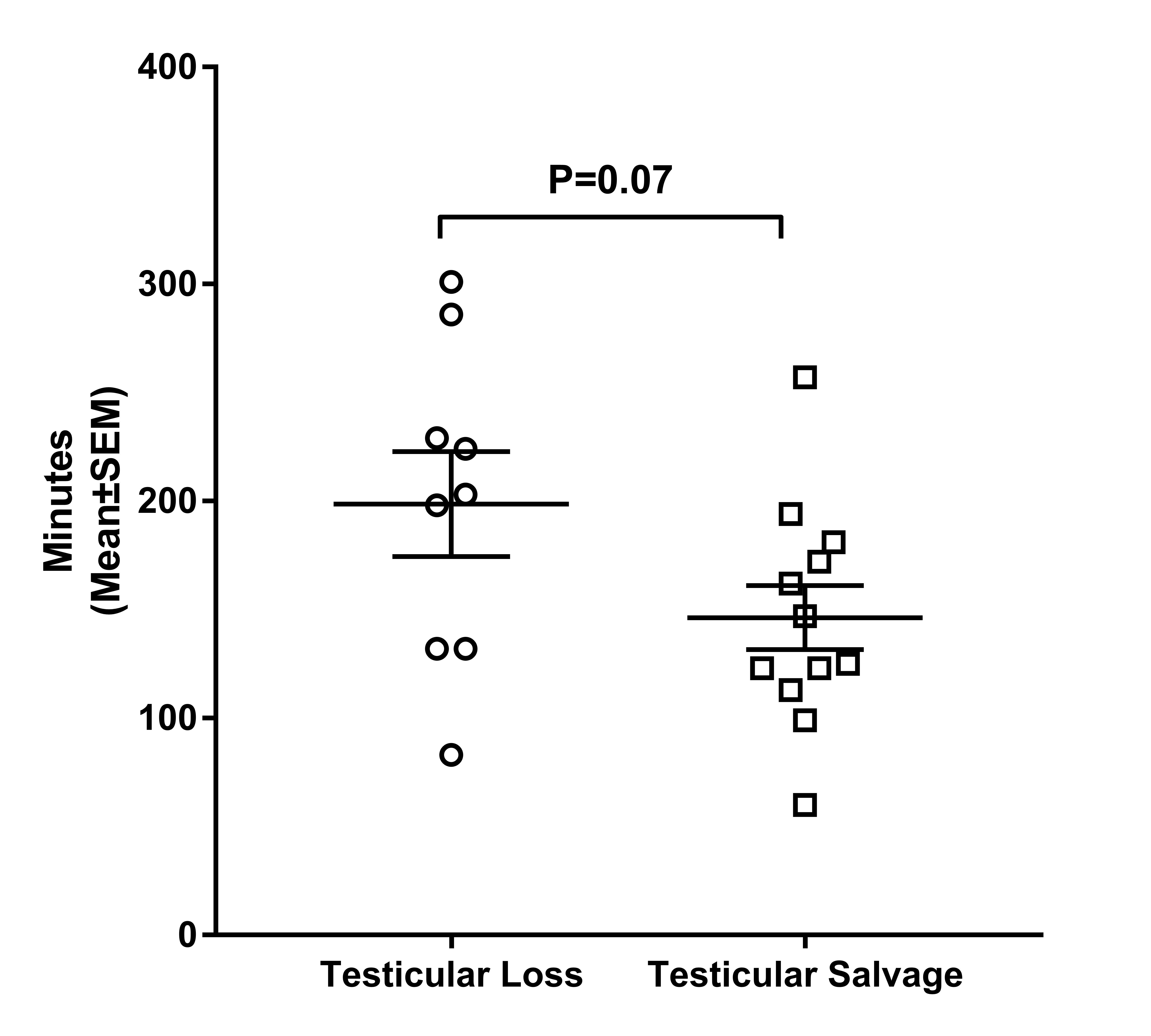

Supplement: Supplementary file 3 [file pqs-4-e232-s003.tif]
